# Supplementary material for: Machine Learning Accelerates Crystallization for Structure Determination
Source: Angew Chem Int Ed Engl. 2026 May 4;65(25):e1218503. doi: 10.1002/anie.1218503 (PMC13266958; doi:10.1002/anie.1218503)

checkCIF/PLATON report

Structure factors have been supplied for datablock(s) lcz018

THIS REPORT IS FOR GUIDANCE ONLY. IF USED AS PART OF A REVIEW PROCEDURE FOR PUBLICATION, IT SHOULD NOT REPLACE THE EXPERTISE OF AN EXPERIENCED CRYSTALLOGRAPHIC REFEREE.

No syntax errors found. CIF dictionary Interpreting this report

Datablock: lcz018

Bond precision:	C-C = 0.0064 A		Wavelength=1.54184
Cell:	a=12.8034(1)	b=14.5922(1)	c=22.2955(2)
	alpha=90	beta=106.249(1)	gamma=90
Temperature:	101 K		

	Calculated	Reported
Volume	3999.07(6)	3999.07(6)
Space group	P 21/n	P 1 21/n 1
Hall group	-P 2yn	-P 2yn
Moiety formula	C15 H3 Ag3 F18 N6, 2(C9 H10 O3)	C15 H3 Ag3 F18 N6, 2(C9 H10 O3)
Sum formula	C33 H23 Ag3 F18 N6 O6	C33 H23 Ag3 F18 N6 O6
Mr	1265.18	1265.18
Dx,g cm-3	2.101	2.101
Z	4	4
Mu (mm-1)	12.944	12.944
F000	2456.0	2456.0
F000’	2467.48	
h,k,lmax	16,18,28	16,18,28
Nref	8599	8166
Tmin,Tmax	0.125,0.143	0.301,1.000
Tmin’	0.023	

Correction method= # Reported T Limits: Tmin=0.301 Tmax=1.000
AbsCorr = MULTI-SCAN

Data completeness= 0.950 Theta(max)= 78.378

R(reflections)= 0.0427(7712) wR2(reflections)= 0.1266(8166)

S = 1.125 Npar= 601

The following ALERTS were generated. Each ALERT has the format
test-name_ALERT_alert-type_alert-level.
Click on the hyperlinks for more details of the test.

Alert level C

PLAT213_ALERT_2_C Atom F00W has ADP max/min Ratio ..... 3.4 prolat
PLAT250_ALERT_2_C Large U3/U1 Ratio for <U(i,j)> Tensor(Resd 1) 2.2 Note
PLAT911_ALERT_3_C Missing FCF Refl Between Thmin & STh/L= 0.600 60 Report
14 0 0, 1 1 0, 0 4 0, 1 4 0, 3 17 0, 1 0 1,
-1 1 1, 14 1 1, 0 2 1, 0 0 2, 14 0 2, -15 1 2,
-1 1 2, 14 1 2, -1 2 2, 14 2 2, 5 16 2, -3 17 2,
-15 0 3, -5 0 3, 1 0 3, 13 0 3, 14 1 3, 14 2 3,
5 16 3, 4 5 5, -1 8 5, -1 10 5, -9 14 5, -2 0 6,
(30 More Missing: see the .ckf listing file)
PLAT913_ALERT_3_C Missing # of Very Strong Reflections in FCF .... 6 Note
0 4 0, 1 4 0, 0 2 1, -5 0 3, -2 2 7, -2 0 8,
PLAT972_ALERT_2_C Check Calcd Resid. Dens. 0.85Ang From Ag02 -1.85 eA-3
PLAT972_ALERT_2_C Check Calcd Resid. Dens. 0.75Ang From Ag03 -1.79 eA-3
PLAT972_ALERT_2_C Check Calcd Resid. Dens. 0.76Ang From Ag02 -1.71 eA-3
PLAT972_ALERT_2_C Check Calcd Resid. Dens. 0.72Ang From Ag01 -1.67 eA-3
PLAT972_ALERT_2_C Check Calcd Resid. Dens. 0.66Ang From Ag02 -1.67 eA-3
PLAT972_ALERT_2_C Check Calcd Resid. Dens. 0.65Ang From Ag03 -1.65 eA-3
PLAT972_ALERT_2_C Check Calcd Resid. Dens. 0.63Ang From Ag02 -1.60 eA-3
PLAT972_ALERT_2_C Check Calcd Resid. Dens. 0.76Ang From Ag01 -1.52 eA-3
PLAT972_ALERT_2_C Check Calcd Resid. Dens. 0.93Ang From Ag01 -1.51 eA-3

Alert level G

PLAT007_ALERT_5_G Number of Unrefined Donor-H Atoms .... 2 Report
H00G H00Q
PLAT083_ALERT_2_G SHELXL Second Parameter in WGHT Unusually Large 7.67 Why ?
PLAT142_ALERT_4_G s.u. on b - Axis Small or Missing .... 0.00010 Ang.
PLAT143_ALERT_4_G s.u. on c - Axis Small or Missing .... 0.00020 Ang.
PLAT242_ALERT_2_G Low 'MainMol' Ueq as Compared to Neighbors of C018 Check
PLAT242_ALERT_2_G Low 'MainMol' Ueq as Compared to Neighbors of C01G Check
PLAT242_ALERT_2_G Low 'MainMol' Ueq as Compared to Neighbors of C01H Check
PLAT242_ALERT_2_G Low 'MainMol' Ueq as Compared to Neighbors of C01N Check
PLAT242_ALERT_2_G Low 'MainMol' Ueq as Compared to Neighbors of C01Q Check
PLAT242_ALERT_2_G Low 'MainMol' Ueq as Compared to Neighbors of C01U Check
PLAT434_ALERT_2_G Short Inter HL..HL Contact F008 ..F00F . 2.84 Ang.
1+x,y,z = 1_655 Check
PLAT434_ALERT_2_G Short Inter HL..HL Contact F00A ..F00A . 2.78 Ang.
1-x,1-y,1-z = 3_666 Check
PLAT720_ALERT_4_G Number of Unusual/Non-Standard Labels .... 89 Note
Ag01 Ag02 Ag03 F004 F005 F006 F007 F008
F009 F00A F00B O00C F00D F00E F00F O00G
H00G F00H F00I N00J F00K O00L N00M N00N

```
              F00O    O00P    O00Q    H00Q    F00R    O00S    N00T    N00U
              N00V    F00W    F00X    C00Y    C00Z    C010    H010    C011
              H011    C012    H012    C013    C014    C015    H015    C016
              C017    C018    C019    C01A    C01B    C01C    H01C    C01D
              C01E    C01F    C01G    C01H    C01I    H01I    C01J    H01J
              C01K    C01L    H01L    C01M    H01H    H01K    H01M    C01N
              C01O    C01P    H01P    C01Q    C01R    H01A    H01B    H01D
              C01S    H01N    H01O    H01Q    C01T    H01E    H01F    H01G
              C01U
PLAT883_ALERT_1_G Absent Datum for _atom_sites_solution_primary ..     Please Do !
PLAT912_ALERT_4_G Missing # of FCF Reflections Above STh/L=  0.600        372 Note
PLAT941_ALERT_3_G Average HKL Measurement Multiplicity ..........        2.5 Low
PLAT969_ALERT_5_G The 'Henn et al.' R-Factor-gap value ..........      4.673 Note
              Predicted wR2: Based on SigI**2  2.71 or SHELX Weight 11.25
PLAT978_ALERT_2_G Number C-C Bonds with Positive Residual Density.          0 Info
```

```
   0 ALERT level A = Most likely a serious problem - resolve or explain
   0 ALERT level B = A potentially serious problem, consider carefully
  13 ALERT level C = Check. Ensure it is not caused by an omission or oversight
  18 ALERT level G = General information/check it is not something unexpected

   1 ALERT type 1 CIF construction/syntax error, inconsistent or missing data
  21 ALERT type 2 Indicator that the structure model may be wrong or deficient
   3 ALERT type 3 Indicator that the structure quality may be low
   4 ALERT type 4 Improvement, methodology, query or suggestion
   2 ALERT type 5 Informative message, check
```

Validation response form

Please find below a validation response form (VRF) that can be filled in and pasted into your CIF.

```
# start Validation Reply Form
_vrf_PLAT213_lcz018
;
PROBLEM: Atom F00W            has ADP max/min Ratio .....        3.4 prolat
RESPONSE: ...
;
_vrf_PLAT250_lcz018
;
PROBLEM: Large U3/U1 Ratio for <U(i,j)> Tensor(Resd    1)        2.2 Note
RESPONSE: ...
;
_vrf_PLAT911_lcz018
;
PROBLEM: Missing FCF Refl Between Thmin & STh/L=    0.600         60 Report
RESPONSE: ...
;
_vrf_PLAT913_lcz018
;
PROBLEM: Missing # of Very Strong Reflections in FCF ....          6 Note
RESPONSE: ...
;
_vrf_PLAT972_lcz018
;
```

```
PROBLEM: Check Calcd Resid. Dens.  0.85Ang From Ag02          -1.85 eA-3
RESPONSE: ...
;
# end Validation Reply Form
```

It is advisable to attempt to resolve as many as possible of the alerts in all categories. Often the minor alerts point to easily fixed oversights, errors and omissions in your CIF or refinement strategy, so attention to these fine details can be worthwhile. It is up to the individual to critically assess their own results and, if necessary, seek expert advice.

PLATON version of 17/09/2025; check.def file version of 17/09/2025

---

duplicate check

No duplication found

Datablock lcz018 - ellipsoid plot

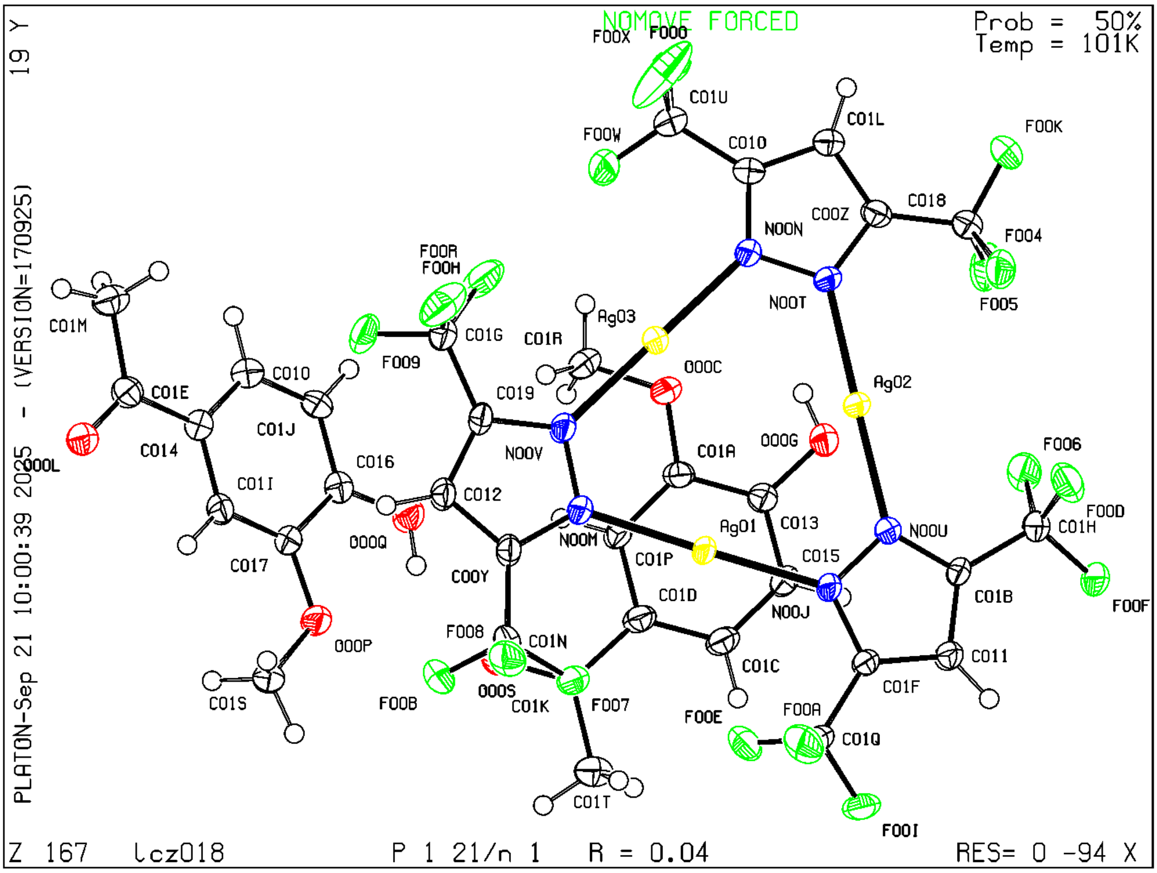

Supplement: Supplementary file 2 — Supporting File 2: anie72462‐sup‐0002‐DataFile.zip. [file ANIE-65-e1218503-s002.zip › supplementary_materials_crystallographic_data submit.zip!/cif_and_checkcif(1-114)/Ag3Pz3_18/Ag3Pz3_18_checkcif.pdf]
